# Mapping HIV-1 Genetic Diversity and Transmission Dynamics in Central African Children: Persistent Challenges and Molecular Complexity

**DOI:** 10.3390/pathogens15101085

**Published:** 2026-10-15

**Authors:** Roseanne Mounanga-Mourimarodi, Gabriel Falque, Jéordy Dimitri Engone-Ondo, Eldridge Fedricksen Oloumbou, Augustin Mouinga-Ondémé

**Affiliations:** 1 Unité des Infections Rétrovirales et Pathologies Associées, Centre Interdisciplinaire de Recherches Médicales de Franceville, Franceville P.O. Box 769, Gabon; roseannemounanga@gmail.com (R.M.-M.); engonejordy@gmail.com (J.D.E.-O.); eldridgefedricksenoloumbou@gmail.com (E.F.O.); 2 Unité Émergence des Maladies Virales, Centre Interdisciplinaire de Recherches Médicales de Franceville, Franceville P.O. Box 769, Gabon; gabfalque@hotmail.fr

**Keywords:** HIV-1, pediatrics, adolescents, PMTCT, antiretroviral therapy, Central Africa

## Abstract

Significant advancements in, and increased access to, antiretroviral therapy (ART) have substantially reduced the risk of mother-to-child transmission (MTCT) of HIV. However, antiretroviral drug resistance (HIVDR) is a serious challenge affecting both adults and children. Because of high HIV prevalence and genetic diversity, Central Africa is considered the epicenter of the epidemic. Yet information on drug resistance profiles and clinical efficacy of newer antiretroviral agents among children is limited compared to adult cohorts. Not to mention that several countries of the subregion, including Gabon, Chad, and São Tomé and Príncipe, lack updated epidemiological data. This article aims to provide an overview of HIV prevention of mother-to-child transmission (PMTCT), focusing on four key elements: prevalence rates; associated risk factors; molecular characterization of pediatric HIV; and virological response to antiretroviral therapy in Central Africa. We have gathered studies published between 2014 and 2026 across PubMed and Research Gate databases and AJOL. The synthesized data reveal high genetic variability of HIV and a dominant CRF02AG variant within the considered populations. The results of this study indicate a range of virological failure rates in the pediatric population on ART for at least six months, ranging from 11.1% to 89.1%. These rates are attributed to a high frequency of resistance-associated mutations (RAMs) and suboptimal adherence to antiretroviral treatment. The main determinants of non-adherence are social stigma, geographic barriers (distance from specialized care centers) and orphan status. Strengthened virological and molecular surveillance programs must be implemented in Central Africa to ensure optimal care for pregnant women, children, and adolescents. Addressing these gaps is imperative in order to bridge the gap between the current epidemiological profile and the WHO’s goals for the elimination of pediatric HIV.

## 1. Introduction

Human immunodeficiency virus (HIV) infection remains a significant global public health concern. As of 2025, it is estimated that 41.0 million people are living with HIV (PLHIV) worldwide, 5.2 million of whom reside in West and Central Africa. Within this regional context, the pediatric population (aged 0–14) is particularly vulnerable, accounting for around 1.3 million children [1]. Despite the intensification of therapeutic scale-up efforts worldwide, 250,000 new infections were reported among children and adolescents under 19 years old in 2024. This highlights a concerning persistence in transmission rates despite the widespread availability of preventive biomedical tools [2,3]. Mother-to-child transmission (MTCT), or vertical transmission, remains the primary cause of pediatric HIV infection [4]. Without clinical intervention, transmission risk ranges from 25 to 40%, occurring during gestation (10–25%), during birth (35–40%), and during breastfeeding (35–40%) [5]. However, the implementation of Prevention of Mother-to-Child Transmission (PMTCT) programs has fundamentally altered this prognosis by successfully reducing residual transmission risk to below 1% through optimized antiretroviral (ARV) coverage for mother–infant pairs [6]. Overall, these interventions have led to a 62% decrease in new pediatric infections between 2010 and 2023 [7]. The survival of vertically infected children is contingent on the timely initiation of antiretroviral therapy (ART). Without pharmacological intervention, infant mortality exceeds 50% by the second year of life [8,9]. In line with the World Health Organization’s (WHO) ‘Test & Treat’ guidelines, the systematic initiation of antiretroviral therapy (ART) upon diagnosis, ideally before the age of three months, is imperative to stop clinical progression. However, infants are disproportionately affected by virological failure and the emergence of resistance-associated mutations (RAMs). These challenges are further exacerbated by late-stage diagnosis, limited access to routine viral load monitoring and prolonged delays in switching to second-line regimens [10,11]. Central Africa occupies a unique position in the molecular history of the pandemic as the Congo Basin has been identified as the zoonotic epicenter of HIV-1 emergence [12]. Although sub-Saharan Africa bears the highest global pediatric burden, the epidemiological and molecular dynamics unique to Central Africa are critically under-documented compared to Southern and Western African regions. To date, there has been no comprehensive characterization of pediatric infection in this region, encompassing prevalence trends, long-term survival rates, viral reservoir dynamics and genotypic resistance landscapes. While the scientific literature provides robust data for adult populations [13,14], systematic evidence regarding children remains fragmented [3,15]. To address this significant knowledge gap, we have conducted a narrative review, synthesizing data published between 2014 and 2025 concerning HIV-1 infection in children and adolescents (aged 0–19) in Central Africa. The review evaluates regional prevalence, the efficacy of localized PMTCT programs, immunovirological responses to ART and antiretroviral resistance profiles. We aim to provide rigorous, evidence-based insights to inform and optimize future public health strategies in the region.

## 2. Search Strategy and Selection Criteria

A comprehensive literature search was conducted in international and regional databases, including PubMed, Google Scholar, ResearchGate, African Journals Online (AJOL), the Owendo Medical Bulletin (BMO), and institutional repositories of gray literature. The objective of this search was to identify articles, institutional reports, and conference abstracts published in English or French between 2014 and 2026.

To ensure the reproducibility of the study, search strings were developed around four main themes, combining terms linked by Boolean operators (‘AND’ or ‘OR’).

Preliminary database searches yielded over 100 documents from PubMed and Google Scholar, 29 from AJOL, and six from BMO. The screening process comprised two stages.

(1) The initial step in the evaluation process is the screening of titles and abstracts. A review of the titles and abstracts was conducted to identify original studies presenting data on PMTCT rates, early infant diagnosis (PCR), viral load monitoring, adherence to antiretroviral therapy, or HIV drug resistance in pediatric populations, children, or adolescents.

The following keywords were utilized:

A comprehensive search strategy was implemented, encompassing prominent bibliographic databases:

- PubMed, Google Scholar, ResearchGate and international databases: “HIV,” “HIV-1,” “child,” “infant,” “adolescent,” “pediatric,” “PMTCT,” “ARV resistance,” “viral load,” “genetic diversity,” and “genotyping.” The following geographical locations were defined for the purposes of this study: “Central Africa,” “Cameroon,” “Gabon,” “Republic of the Congo,” “Democratic Republic of the Congo,” “Central African Republic,” “Chad,” and “Equatorial Guinea.”
- Regional databases (AJOL, BMO and gray literature): equivalent search terms in French were used: The following terms are used in this study: “pediatric HIV,” “children with HIV,” “infant HIV,” “PMTCT,” “mother-to-child transmission,” “Central Africa,” “Cameroon,” “DRC,” “Congo,” “Gabon,” “Chad,” “CAR,” and “Equatorial Guinea.”

(2) Comprehensive evaluation of the text: Subsequently, a full-text evaluation was conducted to ascertain the inclusion of these documents. Studies were excluded if: The following exclusion criteria were applied: (i) the absence of outcome measures specific to the pediatric population; (ii) the exclusive focus on adult or pregnant women cohorts; and (iii) the execution of the study outside Central Africa or non-publication between 2014 and 2026.

In accordance with structured narrative review principles frameworks, a synthesis of eligible studies and high-value gray literature was conducted to address the primary objectives concerning the pediatric epidemiology of HIV, mechanisms of drug resistance, and barriers to care implementation in Central Africa.

## 3. The Prevalence of HIV Infection in Central Africa

Studies on HIV prevalence among infants, children and adolescents are rare in Central Africa. Indeed, few countries from this region have provided data to online databases. Nevertheless, research publications indicate a high incidence of HIV among infants. A study on the prevalence of HIV infection among infants and young children between 2014 and 2024 was published by researchers in Cameroon [16]. Interestingly, the prevalence of vertical HIV transmission appears to reach 22.1% (25/113) among children aged 15 years or under [17]. Although Cameroon shares a southern border with northern Gabon, there appears to be a significant difference in HIV prevalence among children in the two countries. Indeed, a prevalence of 1.0% has been reported among 15- to 19-year-old in Gabon in 2019 [18]. Despite the large number of people tested in this study, the regrettable lack of data on pediatric HIV remains a subject of controversy, as this is likely the only study conducted to estimate the prevalence of HIV among children in Gabon. In Equatorial Guinea, a study conducted after the 2008 update to the national PMTCT protocol reported a prevalence of 26% (14/53) among children aged 18 months and over in 2014 [19]. Although this reported prevalence appears high, the small sample size and lack of confirmatory HIV testing could have significantly influenced this result. In 2024, the use of Xpert HIV-1 Qual (Cepheid) and Geenius HIV-1/2 (Bio-Rad) as confirmatory assays reported an HIV prevalence up to seven times lower than the aforementioned estimate among 54 previously untested HIV-exposed children of a similar age range [20]. In the Democratic Republic of Congo (DRC), Ngwej et al. conducted a study across 12 healthcare facilities in the country’s second-largest city, estimating the rate of vertical HIV transmission at 12.7% (20/157) among infants aged 1.5–16 months [21]. Although this reported prevalence is three to four times lower than the one observed in Cameroon and Equatorial Guinea among infants under two years of age, it is comparable to prevalence reported in Chad in 2015. A previous study reported a prevalence of 11.4% (4/35) among infants with a mean age of two months, whereas another study estimated the prevalence at 14.8% (11/74), but did not specify the mean age of the infants included in the study [22,23]. To our knowledge, only five studies were conducted in Central Africa between 2014 and 2024, covering four of the six countries constituting this region. Despite the significant scarcity of scientific data, the available evidence suggests a high rate of vertical HIV transmission in this region, raising questions about the underlying factors contributing to this mode of transmission.

### 3.1. Routes of Transmission and Factors Involved in the Transmission of Infection in Children

#### 3.1.1. Routes of Infection Acquisition

Mother-to-child transmission (MTCT) remains the primary route of transmission of the infection among children and adolescents, accounting for up to 94.0% of infections among adolescents. This reveals persistent shortcomings in the region prevention programs [24]. Acquisition rates range from 88.5% to 97.2% across cohorts in Cameroon, the Republic of the Congo, and Equatorial Guinea [25,26]. While VT is central, other routes of transmission must not be overlooked. In Equatorial Guinea, parenteral transmission via contaminated blood products and early sexual debut among pre-adolescents (aged 11–13) have been documented [26,27,28,29]. These findings necessitate a high degree of clinical vigilance beyond the perinatal period, in line with WHO observations that, while VT is the main transmission route, pediatric risk profiles are multifaceted. Despite the regional expansion of PMTCT programs, stark disparities in efficacy persist. Significant success has been documented in specialized reference centers in Cameroon and Chad, where incidence rates have decreased to 1.68%, 3.5%, and 5.8% (5/86), approaching elimination thresholds [29,30,31,32]. In other, more recent cohorts of infants, a seropositivity rate of 2.2% (2/91) confirms the persistence of obstetric and pediatric risk factors [33]. However, these successes contrast sharply with unmonitored populations, where transmission rates have been estimated at 89.3%, 90%, and 97.2% in Cameroon, the Democratic Republic of Congo (DRC), and the Republic of Congo (Congo), respectively [26,34,35]. Data from Central African countries diverge significantly from neighboring regions such as Benin, where HIV vertical transmission has been estimated at 3.4%. Such findings highlight the urgent need for healthcare systems to strengthen PMTCT adherence. It is essential that strategies focus on maternal education, socioeconomic support, and the decentralization of antiretroviral treatment administration, with the aim of narrowing the gap between referral centers and more local healthcare services.

#### 3.1.2. Factors Involved in Mother-to-Child Transmission of HIV

It has been demonstrated that maternal viral load (VL) at the time of delivery is the main biological factor in predicting vertical transmission [36,37]. Therefore, the absence of antiretroviral therapy (ART) or delayed initiation of treatment drastically increase the risk of transmission. For example, in the DRC, transmission rates decreased from 45.8% in untreated cohorts to less than 5% when ART was initiated before or during pregnancy [21]. Similarly, in Equatorial Guinea, HIV transmission rates were estimated at 16% (3/19) among children born to mothers who had received ART during pregnancy. By contrast, the prevalence remained higher at 43% (10/23) among those born to mothers who did not receive this treatment [19]. While HIV treatment policies in most Central African countries appear to be led by health authorities, the persistence of high levels of HIV vertical transmission among untreated cohorts appears to be significantly facilitated by a variety of factors, including HIV-related discrimination and late diagnosis. Furthermore, premature rupture of membranes (PROM) is a critical obstetric risk factor associated with transmission rates of up to 80%, increasing the risk of HIV transmission to the newborn by a factor of 45, thereby highlighting the urgent need for standardized intrapartum care [21]. However, PMTCT failure in Central Africa is not only a consequence of pharmacological gaps, but is also deeply rooted in the social determinants of health. Factors such as low educational attainment, widowhood and geographical isolation from healthcare facilities can significantly limit adherence to treatment [16,17,37]. Partner influence also represents a formidable barrier. In Cameroon, for example, it has been demonstrated that widowed women are approximately five times more likely to transmit HIV to their children than married mothers [16]. Moreover, 57% of women report fearing domestic violence following a positive HIV diagnosis [38]. The fear of stigma acts as a profound deterrent to early testing and compromises the achievement of sustained virological suppression, which is essential for preventing vertical transmission. In Cameroon and the DRC, infant feeding practices also play a pivotal role in transmission dynamics. The risk of transmission is significantly higher with mixed feeding (21.4%) than with exclusive breastfeeding or formula feeding (3%) [21,39]. This is attributed to the intestinal inflammation induced by mixed feeding, which compromises mucosal integrity and facilitates the systemic translocation of the virus [40].

### 3.2. Genetic Diversity of HIV in Children

The genetic diversity of HIV-1 within the pediatric population of Central Africa is poorly documented and fragmented due to significant disparities in the literature across countries. Available data indicate a highly variable distribution of Group M clades, characterized by the complex coexistence of pure subtypes and the predominant presence of recombinant forms (Figure 1). Cameroon is the epitome of this genomic complexity. Analysis of pediatric data reveals a complex pattern linking pure subtypes (A1, C, D, F2, G and H) to numerous recombinant variants (CRF09_cpx, CRF11_cpx, CRF13_cpx, CRF22_01A1 and CRF37_cpx). In this viral context, the recombinant subtype CRF02_AG has consistently emerged as the predominant variant, with prevalence ranging from 55.6% to 75.0%, depending on the [11,35,41,42]. CRF02_AG, which is dominant in Cameroon, is also spreading in Equatorial Guinea, accounting for 55.2% of infections among children [28,43]. This epidemiological profile centered on CRF02_AG is consistent with observations made in West Africa, where this variant drives the dynamics of the pediatric epidemic. For example, it accounts for 85.0% of infections in Côte d’Ivoire [44], and dominates the molecular profiles in Sierra Leone, Mali, and Benin [45,46]. It should be emphasized that an “epidemiological silence” persists in Gabon; the complete lack of molecular data among children represents a critical gap in public health information at the regional level. If extrapolations based on data from adults showing a predominance of CRF02_AG serve as a working hypothesis, it is crucial to conduct an initial validation using pediatric cohorts [47,48,49]. The rest of the subregion exhibits unique patterns of divergence. In the Republic of Congo, the epidemic structure is characterized by a high prevalence of unique recombinant forms (URFs, 35.0%), which relegates circulating forms such as CRF45_cpx (10.3%) and pure subtypes G and A3 (8.8%) to a lesser role [50]. In the Democratic Republic of Congo (DRC), pure subtypes (A, A2, B, D, F1, G, H and J) account for the majority of infections (52.8%), compared to recombinant forms (43.6%). Of these recombinant forms, 40.0% consist of URFs and rare CRFs such as CRF27_cpx and CRF47_BF [27]. The Central African Republic continues to exhibit a mosaic co-circulation pattern of pure subtypes (A, C, D and G) and CRFs (CRF01_AE, CRF02_AG and CRF11_cpx) [51,52]. Finally, this regional mapping reveals two other significant knowledge gaps. In Chad, although CRF11_cpx is the predominant genotype in the general population ahead of CRF02_AG, molecular characterizations specific to pediatric cases are lacking. The situation is even worse in São Tomé and Príncipe, where no pediatric molecular typing data are available. The absence of standardized nationwide surveillance in these three countries (Gabon, Chad and São Tomé and Príncipe) severely distorts the modeling of HIV-1 epidemiological dynamics in Central Africa, limiting the optimization of first-line antiretroviral therapies for children in these regions.

## 4. Therapeutic Efficacy and Dynamics of Virological Suppression

### 4.1. Strategies for Combatting the Vertical Transmission of HIV

The systematic administration of antiretroviral therapy (ART) either before conception or during pregnancy, coupled with rigorous postnatal prophylaxis and evidence-based obstetric management (specifically maintaining membrane integrity), is the key to reducing mother-to-child transmission (MTCT). Indeed, studies conducted across Central and West Africa have revealed a direct correlation between early maternal viral suppression and favorable neonatal serological outcomes. In Cameroon, for example, the public health impact of ART is clear: mothers receiving treatment are 2.9 times less likely to infect their babies than those not receiving treatment [57]. Data from Doro et al. (2016) reinforce this finding, reporting residual transmission rates of only 1.5% for ART initiated during pregnancy and a further decline to 0.8% when treatment is established prior to conception [58]. This protective efficacy is mirrored in the DRC, where the contrast in incidence is striking: MTCT rates reach 45.8% in the absence of intervention, compared to 3.1–4.8% among mothers receiving comprehensive ARV coverage [21]. Similar results have been reported in Equatorial Guinea, illustrating the success of PMTCT protocols: in a cohort where the majority of mother–child pairs (approximately 66–69%) received prophylaxis, only two pediatric infections were confirmed [59]. In other words, this study’s results show a mother-to-child transmission rate of 2.9% (2/69), which is approximately 0.5 times higher than in Cameroon and 0.6–0.9 times lower than in the DRC. However, a significant gap remains in the regional literature. The available data, particularly the proportion of mother–child pairs showing persistent HIV transmission despite treatment, raise questions about the causes of this observation. Is the phenomenon solely related to treatment adherence, or are viral or molecular factors intrinsic to the infecting virus also involved?

### 4.2. Mapping of the Virological Response to Antiretroviral Treatment

Current data on pediatric HIV highlight a significant age-related difference in treatment efficacy. A multicenter study conducted in Cameroon highlighted the increased vulnerability of pediatric populations, revealing viral suppression (VS) rates of 90.8% (7799/8585) among adults aged 20 and older, 74.4% among adolescents (ages 10–19), and an alarming rate of 64.8% among children (ages 0–9) [60]. A few years later, similar results were obtained in Cameroon [29]. As a result, children and adolescents are significantly more likely to experience virologic failure (VF) than adults. This observation is supported by data reported from Equatorial Guinea. In that country, virologic failure (VF), defined as a viral load (VL) greater than 1000 copies/mL, reaches alarming levels among children (84.2% [32/38]) and adolescents (88.9% [16/18]), whereas it affects only 61.9% [70/113] of adults [27]. The same observation was first made in Gabon, where a study of children and adults found the following percentages: children aged 0–7: 23.28% (5/21); 29.2% (14/48) among those aged 8–15; and 42.6% (26/61) among those aged 16–24 [61].

One of the main factors explaining this disparity is unequal access to next-generation antiretrovirals.

The shift to dolutegravir (DTG)-based treatment in Equatorial Guinea is associated with significantly higher viral suppression (VS) rates (91.9%) compared to treatments using non-nucleoside reverse transcriptase inhibitors (NNRTIs) (86.4%) or protease inhibitors (PIs) (59.7%) [28]. This observation was made during a study designed to evaluate the clinical and virologic efficacy of treatment regimens containing DTG and efavirenz (EFV) in children and adolescents. After 18 months of treatment, the results showed that 100% of patients on DTG had achieved viral suppression, compared with 91.7% of those on EFV [62]. Comparable results were obtained in the Republic of Congo and Cameroon in 2026, where viral suppression was much more strongly associated with the use of a dolutegravir (DTG)-based antiretroviral therapy (ART) [63,64] Studies have reported improved clinical outcomes with the use of DTG in children living with HIV, including an increase in viral suppression rates:

- In Nigeria, treatment outcomes for 180 infected children who switched from an LPV/r-based regimen to DTG showed that viral suppression rose from 66% at baseline to 90% after 12 months.
- In 2024, in South Africa, 50% of children living with HIV under the age of ten on DTG achieved viral suppression, with rates ranging from 64% to 73% [65].

However, the use of DTG in pediatrics remains limited: only 1.2% (82/7117) of children and 1.9% (138/7117) of adolescents are receiving DTG, whereas 96.9% (6897/7117) of the adult population in Cameroon is using it [60]. Statistics from Lesotho (2024) reveal that after switching to DTG, viral suppression rates could rise to 91.8% (1846/2012) within a year, underscoring the urgent need for a rapid transition to integrase strand transfer inhibitors (INSTIs) [66].

A 2026 study conducted in the Republic of Congo found that 34% (173/510) of participants had a detectable viral load (>40 copies/mL) while on antiretroviral treatment [63].

Furthermore, elevated rates of uncontrolled viremia and therapeutic failure have been documented among cohorts with confirmed HIV status undergoing diagnosis. In Equatorial Guinea, for example, levels of uncontrolled viral load (above 1000 copies/mL) have been reported to range from 82.9% to 90% among children and adolescents undergoing antiretroviral therapy [20]. While these figures represent a strictly selected high-risk group rather than the entire pediatric population analyzed, they nonetheless emphasize significant gaps in systematic virological monitoring and the rapid optimization of treatment regimens. In Cameroon, confirmed virological failure (defined as two consecutive viral load measurements of ≥1000 copies/mL) was observed in 11.1% (31/280) of patients, while 9.6% (27/280) exhibited low-level viremia (200–999 copies/mL) [67]. In Equatorial Guinea, 89.1% of children and adolescents exhibited virological failure at the time of sampling, despite a median duration of antiretroviral treatment duration of six years and a median viral load of 11,824 copies/mL [27]. Furthermore, geographical disparities persist, with satisfactory viral load monitoring coverage in urban centers (84%) but deficient coverage in rural areas (37%), likely reflecting disparities in healthcare access and broader sociodemographic inequalities [68]. These disparities may contribute to the disproportionately high rates of virological failure observed in Central Africa, highlighting the need for further research into the underlying causes.

The failure rates observed in Central Africa stand in stark contrast to the relative successes seen in West Africa. For example, the virological failure in Togo was 18.2% in 2018. Indeed, a study conducted at the Sylvanus Olympio University Hospital in Togo reported a virological failure rate of 18.2% (viral load > 1000 copies/mL) among children and adolescents on ART for at least of 12 months, with a median treatment duration of six years [69].

### 4.3. Determinants of Virological Failure: Adherence Dynamics and Selection Pressures

The response to antiretroviral therapy (ART) among children and adolescents in Central Africa varies according to the therapeutic regimens employed and the quality of longitudinal clinical follow-up. Although continent-wide studies [70,71,72] report encouraging virological suppression (VS) rates, Central Africa continues to face persistent challenges in achieving the UNAIDS 95-95-95 targets.

#### 4.3.1. Poor Adherence to Antiretroviral Treatment

Sustained adherence to treatment is essential for viral suppression and reducing morbidity and mortality. In Central Africa, data reveal systemic weaknesses in adherence. Indeed, rates of non-adherence as high as 41.0% (80/195) have been documented among adolescents (aged 16.8 ± 1.5 years) in Cameroon [73]. Of particular concern is the gradual decline in adherence observed among adolescents aged 10 to 19 who were infected perinatally. The fact that adherence is associated with virological failure following perinatal HIV infection is a serious cause for concern. In other words, adherence to antiretroviral therapy has declined over time: from 67.3% (37/55) at enrolment, to 51.0% (49/96) at six months, and to 50.0% (30/60) at 12 months, across three successive follow-up periods. This highlights the considerable challenges in maintaining treatment adherence among this vulnerable population [58]. Similarly, although adherence rates ranging from 48% to 54.9% have been reported in the Republic of Congo, these levels remain insufficient to ensure sustained viral suppression [26,74]. Although data on ART adherence appear to be lacking in other Central African countries, the similarities observed in mother-to-child HIV transmission, virological failures and adherence rates in the region may reflect common sociodemographic and health conditions. This suggests that there are common multifactorial determinants of adherence. The drivers of ART adherence in Central Africa can be categorized into three critical domains that directly influence viral kinetics and long-term clinical outcomes.

#### 4.3.2. Intrinsic and Demographic Predictors

Age remains a primary predictor of therapeutic compliance. Higher levels of adherence are generally documented in pre-adolescent children (under 15 years old), where treatment is typically overseen by a primary caregiver [74]. Conversely, children under 5 years of age are less likely to achieve viral suppression [75]. Male sex appears to be an independent predictor of viral suppression [76]. with lower suppression rates observed in males than in females (78.2% vs. 85.8%, *p* < 0.001) [75]. Furthermore, some Congolese studies have reported a gender-based trend, with slightly higher adherence rates observed among male pediatric patients [11,26].

#### 4.3.3. Psychosocial and Environmental Social Determinants

The family unit and social integration are the main foundations for therapeutic success.

- Orphanhood: The loss of one or both parents is a critical risk factor for non-adherence, often due to household destabilization and loss of consistent treatment supervision [77].
- Geographic disparities and monitoring systems: Viral suppression rates are lower among patients living in rural areas, than among those living in urban areas (40.48% vs. 56.96%). This represents a disadvantage for these populations [78]. Furthermore, receiving care at decentralized health centers increases the risk of virological failure by a factor of 1.76 compared to accredited centers of excellence (*p* < 0.001) [79]
- Protective clinical factors: Strong relationships with healthcare providers, the active involvement of guardians and participation in peer-led support groups are essential protective factors that bolster resilience and retention in care [25].

#### 4.3.4. Factors Related to ART

Tolerance to antiretroviral therapy (ART) directly influences adherence patterns. The incidence of adverse drug reactions (ADRs), the cumulative ‘pill burden’, and the exhaustion associated with lifelong exposure (particularly for individuals who started treatment in early infancy) are strongly correlated with suboptimal adherence [73,80]. However, it is important to note that virological failure (VF) should not be viewed solely in terms of patient behavior. The emergence of drug resistance mutations (DRMs) resulting from prolonged exposure to sub-therapeutic drug levels constitutes a formidable biological barrier. Once established, these mutations compromise the regimen’s efficacy regardless of subsequent improvements in adherence, necessitating robust and routine genotypic surveillance to guide therapeutic switching.

#### 4.3.5. Factors Specific to Different Pediatric Populations

Factors specific to children (<10–12 years):

Starting antiretroviral therapy (ART) at an advanced clinical stage of the disease (WHO stages III/IV) has been shown to strongly predict a persistently high viral load [81]. Added to this is the efficacy of the treatment regimens used [24].

Factors specific to adolescence (ages 12–19):

Conversely, adolescence is a vulnerable phase; this developmental transition is often associated with treatment fatigue, struggles for autonomy, and an increased risk of treatment discontinuation [11]. Stigma and discrimination: Internalized stigma is a formidable structural barrier that severely undermines psychosocial well-being and discourages the use of health services [80]. Clinical protective factors: Strong relationships with healthcare professionals, active involvement of guardians, and participation in peer-led support groups are essential protective factors that enhance resilience and ensure the continuity of care [26]. In 2024, it was reported that low self-esteem and a reduced quality of life were risk factors for poor adherence to antiretroviral therapy and an uncontrolled viral load among adolescents [82].

## 5. Genotypic Profile and Emergence of Drug Resistance Mutations (DRMs)

Molecular surveillance of antiretroviral (ARV) resistance among children in Central Africa is still in its infancy. Between 2017 and 2024, fewer than ten studies have addressed this issue. These studies are concentrated in only four countries in the subregion: Cameroon (nearly five), the Central African Republic (two), the Democratic Republic of Congo (one), and Equatorial Guinea (one). This highlights a critical ‘molecular gap’, as no recent data are available for Gabon, Chad, and the Republic of Congo.

The available data show that there are remarkably high rates of drug-resistant mutations (DRMs) in pediatric patients who have experienced virologic failure, ranging from 45.0% in the Central African Republic to 100% in certain Cameroonian patients group [11,42,67,83]. In the Central African Republic, for instance, non-adherence to antiretroviral treatment (ART) accounted for 97.5% of treatment failures in one of the subgroups studied in 2020, while pre-treatment resistance in infants who had never received treatment reached 52.6%. [84]. The relevant literature indicates that there is a high degree of similarity in the prevalence rates of major DRMs are highly similar in the Democratic Republic of the Congo (67.3% in 2021) and Equatorial Guinea (62.8%) [27,28]. The elevated levels of resistance documented in several Central African studies are evidently substantially influenced by cumulative selection biases. In practice, resistance testing in the region, is often delayed and reserved for patients with chronic and prolonged virologic failure (lasting more than one to two years), rather than for those with confirmed early non-suppression in the short term (e.g., within four weeks). Furthermore, the variability in the success of the sequencing process, as evidenced by numerous studies, frequently results in a substantial reduction in the number of samples. This constrains the ultimate estimates of resistance mutation rates within the study population to a limited subset of samples that are amenable to amplification or sequencing. The combination of prolonged selective drug pressure and the technical selection of samples has been shown to lead to an increase in resistance mutation rates in samples that undergo genotyping.

### 5.1. Resistance to ART Before Transitioning to a DTG-Based Regimen

Resistance to reverse transcriptase inhibitors (NRTIs and NNRTIs)

Almost all molecular resistance profiles are dominated by treatment failures in first-line regimens based on NRTIs and NNRTIs. This epidemiological pattern is highly consistent with data from West Africa (Togo, Côte d’Ivoire and Sierra Leone) [44,45,69].

- Non-Nucleoside RT inhibitors (NNRTIs): Among children with treatment failure, NNRTIs demonstrate the highest rates of resistance, ranging from 40.7% to 95.4% in Equatorial Guinea, the Central African Republic (CAR), Cameroon and in the Democratic Republic of the Congo (DRC) [28,42,83,85]. More than half of the children receiving first-line therapy had mutations conferring resistance to first-generation NRTIs, particularly the K103N mutation (52.3%). V106M, Y181C, and Y188H substitutions were also observed [67,83,86,87].
- Nucleoside RT inhibitors (NRTIs): Resistance to NRTIs follows a similar trend.

In cases of treatment failure, resistance to NRTIs is frequently observed, reaching up to 90.9% among treated children in Equatorial Guinea, in the DRC and in the CAR [67,85,86]. Mutations in thymidine analogs (35.7%) affect zidovudine, while the M184V mutation predominates (47.6%), conferring high resistance to lamivudine [11]. Other profiles include the K65R, T215I, and K219E mutations, each of which was observed in 33.0% of cases [83].

Resistance to protease inhibitors (PIs) and integrase inhibitors (INIs)

Resistance to IPs remains moderate, ranging from 18.2% to 27.6% depending on the cohort. Most of cases involve minor mutations [83,85]. However, major resistance to PIs was found in 24% of children who failed first-line treatment, major resistance to protease inhibitors (PIs) was found [87]. With regard to susceptibility to INSTIs, this remains largely preserved (in the majority of children, 88.9%); the mutations observed are primarily natural polymorphisms or accessory mutations [11,83,85].

### 5.2. Resistance to TAR Following Transitioning to a DTG-Based Regimen

In the context of transitioning to a DTG-based regimen, virological failure in the pediatric population was associated with a high level of resistance. The mutations primarily target NNRTIs and NRTIs.

Non-Nucleoside RT Inhibitors (NNRTIs): Between 40.7% and 73.5% of the population harbors NNRTI-resistant viruses. The main mutations associated with NNRTI resistance are K103N, V106M, Y181C, and Y188H [83]. The M184V mutation (53/98, 54.1%) was the most prevalent NRTI-associated mutation in RC [63].

Nucleoside RT inhibitors (NRTIs): Resistance-conferring mutations to NRTIs were present in 3.7% to 61.2% of the pediatric population. The main mutations were M184I/V, K65R, T215I, and K219E [27,83]. Dual resistance to NRTIs/NNRTIs was observed in 53.1% of individuals [27]. In the Republic of Congo, in 2026, mutation was the most common mutation associated with NNRTI resistance was K103N (40/98, 40.8%) [63].

These data are consistent with the observations made by Afrane et al. in Ghana, where all participants (100%, 20/20) had NNRTI resistance mutations and 75% (15/20) had NRTIs resistance mutations. The mutation profiles were predominantly characterized by the M184I/V (75%, 15/20) and K103N (60%, 12/20) mutations [88].

High rates of resistance to several currently used drugs were observed. In particular, resistance to 3TC, and the NNRTI drugs EFV (62%) and NVP (72%). The observed protease resistance mutations (RAMs) were significantly associated with prior use of LPV/r, as well as with major RAMs (*p* = 0.018). Exposure to EFV was also significantly associated with NNRTI resistance mutations (*p* = 0.019) [63].

Resistance to protease inhibitors (PIs) and integrase inhibitors (INIs):

Resistance to PIs remains moderate (8.3% to 27.6%) and consists mainly of minor mutations [27,83]. With regard to INSTIs, overall, it has generally been reported that susceptibility to INSTIs remains very well preserved [63]. Only minor accessory mutations are present. However, analysis of INIs reveals mutation rates of 11.1% in the Central African Republic (2019) and 10.7% in Cameroon (2023) [35,51], while the DRC reported 15.0% minor mutations in 2021. At the molecular level, the E138K/T and G140R substitutions were identified. According to the Stanford HIV drug resistance database algorithm, the E138K/T substitution confers low-level resistance to raltegravir (RAL) and elvitegravir (EVG), with a potentially minimal impact on dolutegravir (DTG); meanwhile, the G140R substitution impairs the efficacy of cabotegravir (CAB). While no major, high-level resistance directly compromising DTG has yet been confirmed in Central Africa, the recent identification of 9.4% prevalence of major INSTI resistance among children in Togo [89] highlights the urgent need for intensified epidemiological surveillance of DTG resistance throughout the subregion. The levels of DTG resistance reported in Central Africa are lower than the figures reported by the WHO in 2024. However, WHO surveys have reported the emergence of HIV resistance to DTG at higher levels, ranging from 3.9% to 8.6% and reaching 19.6% among individuals with a long treatment history. Therefore, it is imperative for countries in Central Africa to implement standardized surveillance of HIV resistance to antiretrovirals among people with detectable viral loads, as recommended by the WHO [90].

### 5.3. Prevalence and Molecular Profile of Pre-Treatment Resistance (PTR) in Treatment-Naïve Children

Assessing pre-treatment resistance (PTR) in children who have never received antiretroviral therapy (ART) is a neglected aspect of virological surveillance in Central Africa. To date, only one study has documented this issue, conducted in Cameroon in 2020 [85]. The near-total absence of molecular data at the subregional level means it is not possible to create an accurate map of the transmission of resistant variants among infants and young children. Despite its small sample size, the Cameroonian study reveals a concerning epidemiological situation. The overall prevalence of drug resistance (PDR) among these children was 52.6% (10/19) [85]. A thorough review of medical histories highlights the direct effects of the Prevention of Mother-to-Child Transmission (PMTCT) program. Among children exposed to PMTCT, failure of neonatal prophylaxis frequently leads to the emergence of resistant variants. Notably, 57.1% (4/7) of children not exposed to PMTCT who had never received antiretroviral prophylaxis exhibited major mutations associated with ART resistance, confirming direct vertical transmission of resistant strains from mother to child [85]. Analysis by drug class revealed decreased viral susceptibility affecting all molecules used in pediatrics during this period: 31.6% (6/19) for NNRTIs (dominated by the Y181C mutation); 26.3% (5/19) for NRTIs (major mutation L174V/I); and 15.8% (3/19) for PIs (mutations M46I, V23I and D30N) [85]. These high rates of PDR in Central Africa are consistent with observations in other regions with limited resources, confirming that transmitted resistance in treatment-naïve children is a global health crisis. In West Africa, data from Nigeria reveal a pattern that closely mirrors that of Cameroon. A study examining drug resistance in children before and after treatment found that 48.0% (10 out of 21) of cases carried major resistance mutations. However, among these resistant patients, the majority (8 out of 10 children) had never been exposed to ARVs [91]. This finding corroborates the observations of Dambaya et al., confirming the extent to which resistant variants are transmitted vertically by mothers experiencing treatment failure. This vulnerability has also been observed in treatment-naïve children outside of Africa, particularly in Southeast Asia. In Vietnam, a study of infants under 18 months of age revealed a resistance rate of 43.8% [92]. Consistent with the mutation profiles of pediatric treatment regimens, resistance to the NNRTI class was predominant, accounting for 37.7% of cases.

## 6. Diagnostic Delays and Latency in Therapeutic Initiation

According to the WHO, it is crucial to rapidly identify HIV in children and initiate antiretroviral therapy (ART) without delay to prevent irreversible immune system deterioration and reduce child mortality [93]. Despite these guidelines, Central Africa faces enormous challenges in pediatric care, characterized by delayed diagnoses and significant delays before treatment begins. In Equatorial Guinea, limitations persist between screening and treatment. An initial study identified delays in diagnosis in 8.0% of children and 21.4% of adolescents, as well as delays of over one year between diagnosis and the commencement of ART in 13.5% of children and 17.6% of adolescents [28]. More recent data from Guinea corroborate these findings: the median age at diagnosis was nine years for children and 13 years for adolescents, with a delay in starting ART exceeding one month for 12.5% of children and 36.8% of adolescents [20]. Late diagnosis leads directly to clinical manifestations of advanced disease. In Cameroon, a study found that over half of children (52.5%) were diagnosed at an advanced stage of the disease [34]. An even more alarming situation has been reported in the Democratic Republic of Congo (DRC), where 75% of children living with HIV receiving care at Heal Africa hospitals were at WHO Stage 3 at the time of diagnosis [25]. Similarly, rates of delayed diagnosis of up to 74.2% and delays in treatment initiation of 83.3% were also observed in the DRC [94]. This care pathway has a direct consequence of significantly limiting the early onset of therapeutic efficacy [94].

These findings highlight the urgent need to improve active and early screening strategies in this subregion.

## 7. Analysis of Mortality in Central Africa

The infant mortality rate peaks during the first two years of life, whether due to inadequate treatment or insufficient adherence to antiretroviral therapy (ART) among pregnant women [95]. However, a review of the literature reveals a significant disparity in epidemiological data within the subregion. This disparity is characterized by a concentration of studies conducted in Cameroon, in contrast to a near-total lack of published molecular and virological data for neighboring countries such as the Central African Republic, Gabon, the Republic of Congo, Chad and Equatorial Guinea. In Cameroon, mortality statistics among children infected with or exposed to HIV vary considerably depending on time period, treatment status and place of residence. According to an observational study conducted in Cameroon in 2015, the overall mortality rate was 15.4% (44/285), with a significantly higher incidence in rural areas during the period from 2004 to 2012 [96]. Temporal and methodological variations are observed in modern urban cohorts: For example, Tejiokem et al. reported a mortality rate of 5.7% (12/210) among infants [97], while Doro et al., in 2016, observed a one-year survival rate of 92%, which corresponds to a mortality rate of 8% (22/275) [58]. The impact of early therapeutic intervention on pediatric survival is crucial. In Cameroon, in 2016, Nguefack et al. documented an alarming infant mortality rate of 34.7% (52/150) among those who were not screened or received late treatment (median age at death: seven months) [98]. In this study, the survival rate among children on antiretroviral therapy (ART) was 84.3%, compared with 43.3% among those who were not treated (*p* = 0.05), demonstrating the direct prognostic role of treatment initiation [85]. By contrast, the significant decrease in mortality to 2.58% (28/1086), as reported by Njom Nlend et al., is associated with prompt diagnosis. Indeed, 94.4% of children in this cohort were categorized as WHO Stage I at enrolment [98]. This close correlation between delayed diagnosis and infant mortality forms part of a broader pattern observed in sub-Saharan Africa and South Asia [99]. Similar epidemiological patterns have been observed in other countries with a high disease burden, such as India, where HIV-related pediatric mortality was reported at 3.4% in 2014 [100], and in Uganda, where rates ranged from 3.9% to 12.4% in 2012 [101]. Taken together, these data highlight the urgent need for policymakers to implement systematic strategies for early virological diagnosis at birth, enabling the immediate initiation of antiretroviral therapy (ART).

### Causes and Determinants of Infant Mortality

Recent multicenter data from the CHAMPS project [99] have identified HIV as one of the main infectious factors associated with early infant mortality. In the DRC, the causes of mortality and morbidity among children living with HIV depend on a combination of opportunistic clinical manifestations and adverse maternal and infant biological indicators. The main causes of hospitalization documented in this population are primarily opportunistic respiratory infections, followed by prolonged febrile syndromes of undetermined origin, severe malnutrition, anemia and acute diarrheal diseases [34]. In Cameroon, a lack of antiretroviral prophylaxis or an insufficient initiation of combination antiretroviral therapy (cART) is consistently associated with a rapid progression to advanced WHO stages [102]. Indeed, antiretroviral therapy (ART) significantly influences survival rates among infected infants. In 2016, survival rates were higher (84.3%) among infants receiving ART than among untreated infants (*p* < 0.05) [85]. Although some cohorts show a lower overall mortality rate (2.58%), survival studies in Cameroon highlight significant variations depending on the feeding method [83]. Mixed feeding, which combines breast milk with other food substitutes, exposes infants to a 5.4- to 5.5-fold higher risk of mortality than exclusive breastfeeding due to impairment of the intestinal mucosal barrier which facilitates systemic transmission of the virus [98]. Beyond clinical manifestations alone, previous studies conducted outside the subregion have reported that the kinetics of viral replication and the severity of immunosuppression are the main predictors of early infant mortality. Quantitative data indicate that a high plasma viral load in both infants and their mothers is associated with a significant increase in the risk of death, with risk ratios (RRs) of 8.54 and 2.50, respectively [95]. From an immunological perspective, a maternal CD4 count below 200 cells/mL is an independent predictor of infant mortality (hazard ratio (HR) = 2.61). This accelerated progression in infants is strongly correlated with severe maternal immune deficiency at the time of gestation or delivery [103]. The impact of viral load is such that, in the absence of immediate treatment, 67.6% of children with viral loads above the cohort median die before the age of two [95].

## 8. Persistent Challenges and Molecular Complexity: Structural Scarcity of Local Genotypic Data

A review of the literature reveals a shortage of molecular data on pediatric HIV in Central Africa due to the structural scarcity of local genotypic data. This scarcity stems from the material constraints of RT-PCR and HIV genotyping sequencing platforms in sub-Saharan Africa. These include a lack of appropriate infrastructure, high reagent costs, centralization of reference laboratories and maintenance issues [104,105], as these factors depend on temporary external funding, the sustainability of autonomous, continuous epidemiological surveillance is hindered [104].

Consequently, pediatric care in Central Africa still primarily relies on clinical and immunological monitoring, despite global WHO recommendations regarding the surveillance and monitoring of ARV resistance [106,107] resistance. Recognizing this limitation is a critical finding in itself, underscoring the urgency of strengthening local diagnostic capacities [108].

Clinical and Epidemiological Challenges Posed by the Lack of genotyping.

This lack of pediatric genomic surveillance creates two major challenges in Central Africa:

(1) Accumulation of resistance through blind treatment: Pediatric exposure to ARVs during pregnancy or through breastfeeding promotes the emergence of transmitted resistance mutations. Without accessible genotyping tests, second- or third-line treatment choices can not be guided by genetic information, with increases the risk of virologic failure and multidrug resistance [105].
(2) Impact of regional viral hyperdiversity: Central Africa, the birthplace of HIV-1, exhibits a unique genetic mosaic dominated by complex recombinant forms and multiple subtypes. The rarity of routine sequencing prevents the assessment of the long-term efficacy of newer drugs (such as pediatric dolutegravir) from being assessed in the face of this genetic diversity.

## 9. Conclusions

This review highlights the complexity of the epidemiological, clinical, and molecular dynamics that characterize HIV infection among children in Central Africa. Despite the implementation of programs to prevent mother-to-child transmission (PMTCT), the prevalence of infection and associated mortality remain high in the subregion. The effectiveness of PMTCT interventions is undermined by various structural and behavioral constraints, including a lack of treatment information among women of childbearing age, social stigma, suboptimal adherence to antiretroviral therapy (ART) and limited access to healthcare services in rural areas. Therefore, optimizing therapeutic education appears to be a key way of maximizing the impact of these preventive programs. From a virological perspective, Central Africa is characterized by exceptional genetic heterogeneity and variability, dominated by the circulation of recombinant forms, primarily CRF02_AG. This complexity is accompanied by alarming rates of virological failure, which are strongly associated with the emergence of high-level resistance mutations against nucleoside reverse transcriptase inhibitors (NRTIs) and non-nucleoside reverse transcriptase inhibitors (NNRTIs). The trajectory of virologic failure and loss to follow-up is exacerbated among the particularly vulnerable population of adolescents by multifactorial determinants such as age-related transitions, sexual dynamics, the degree of parental involvement, the quality of the relationship with healthcare providers, and treatment fatigue associated with the duration of antiretroviral therapy (ART). In summary, the data compiled in this review highlight the urgent need for clinicians and health authorities to systematically monitor viral load and integrate genotypic resistance testing into routine care. The key strategy for curbing the spread of multidrug resistance is this transition towards molecular profile-guided care.

As recommended by the WHO, surveillance studies are essential for several countries in Central Africa. Indeed, the provision of optimal care for pregnant women, children, and adolescents in this subregion is compromised by numerous factors. These include, among many others, the distance between patients’ homes and health centers, shortages of antiretroviral (ARV) drugs, screening and early diagnosis kits (Polymerase Chain Reaction (PCR)), patients’ socioeconomic conditions, and the phenomenon of patients lost to follow-up.

## Figures and Tables

**Figure 1:**
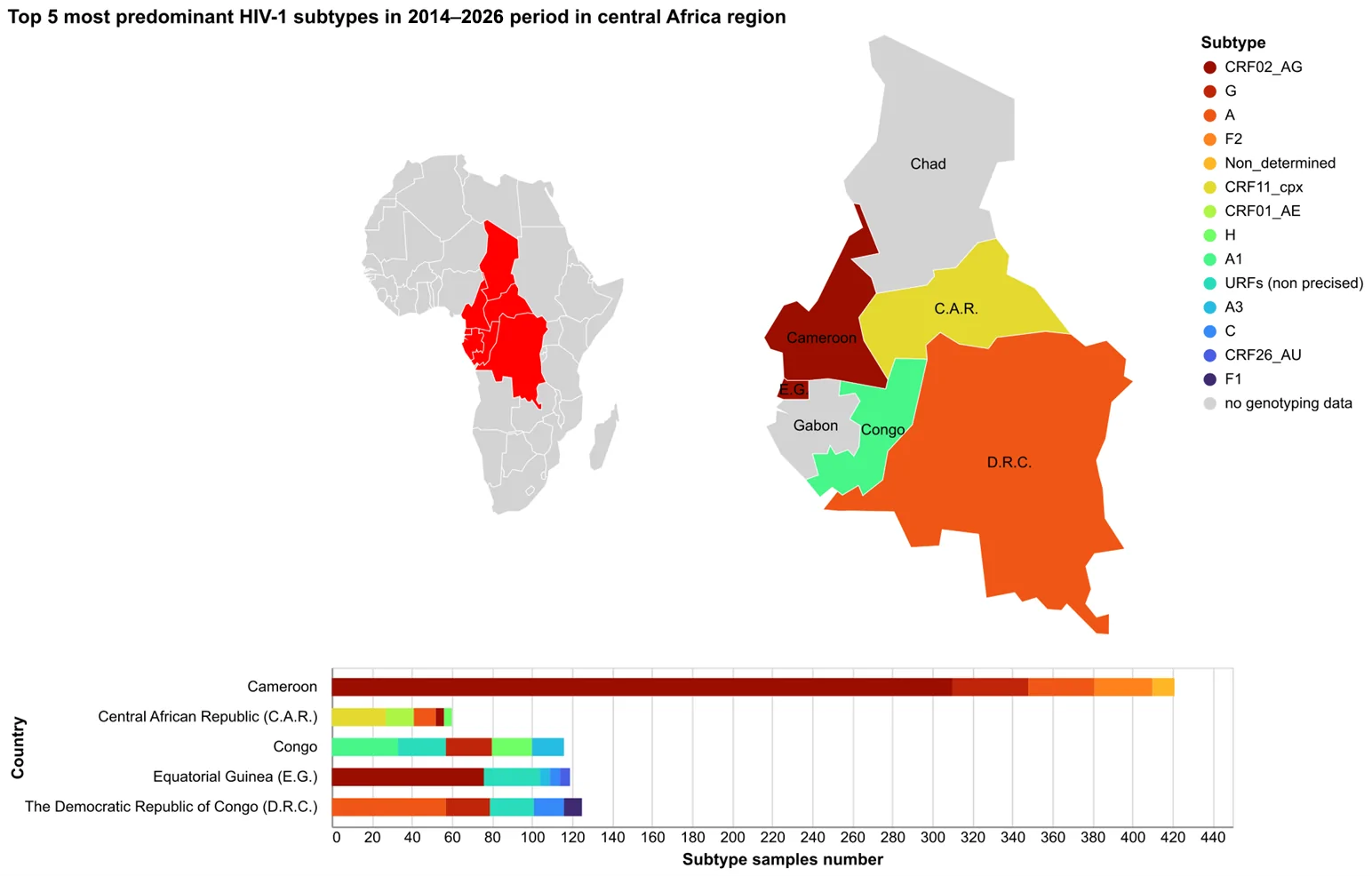
This map illustrates the prevalence and distribution of HIV-1 clades and pediatric genotypes in Central Africa between 2014 and 2026. The map at the top illustrates the predominant variant identified in each country. It reveals that the CRF02_AG recombinant is dominant in the northern and coastal parts of the region, while unique recombinant forms (URFs) prevail in the Congo Basin (DRC). Gray areas indicate a lack of published pediatric molecular data for the period in question. The bar chart at the bottom shows the quantitative distribution of the five most common genotypes by country. The data summarize the results of ten independent genotyping studies (selected from sixty identified publications). Abbreviations: URFs: unique recombinant forms; E.G.: Equatorial Guinea; D.R.C.: Democratic Republic of Congo; C.A.R.: Central African Republic. The data were analyzed using Python version 3.12.12 [53] in a Jupyter Notebook [54] with the Pandas package version 3.0.6 [55], and the graphs were generated using the Altair package version 6.3.0 [56].

## Data Availability

The authors confirm that all relevant data are within the paper. For researchers who meet the criteria for access to confidential data, the data are available on request from the corresponding author.
